# Analysis of Honeybee Drone Activity during the Mating Season in Northwestern Argentina

**DOI:** 10.3390/insects12060566

**Published:** 2021-06-21

**Authors:** Maria Marta Ayup, Philipp Gärtner, José L. Agosto-Rivera, Peter Marendy, Paulo de Souza, Alberto Galindo-Cardona

**Affiliations:** 1 National Scientific and Technical Research Council, CONICET, CCT, Tucumán 4000, Argentina; martitaayup@gmail.com; 2 Faculty of Natural Sciences, National University of Tucumán (UNT), Tucumán 4000, Argentina; 3 IER (Regional Ecology Institute), CONICET, Tucumán 4000, Argentina; gaertner.p@gmail.com; 4 Rio Piedras Campus, University of Puerto Rico, Rio Piedras 00927, Puerto Rico; jose.agosto1@uprrp.edu; 5 Commonwealth Scientific and Industrial Research Organisation, CSIRO, Canberra 2601, Australia; peter.marendy@csiro.au (P.M.); paulo.desouza@griffith.edu.au (P.d.S.); 6 School of Technology, Environments and Design, University of Tasmania, Tasmania 7000, Australia; 7 School of Information and Communication Technology, Griffith University, Nathan 4111, Australia; 8 Miguel Lillo Foundation, Tucumán 4000, Argentina

**Keywords:** drone activity, drone congregation areas, mating behavior, RFID technology, video recording

## Abstract

**Simple Summary:**

When we think of social insects, we mostly neglect males and their biology. Honeybee males or drones are an example. Like queens, drones are founders of the colony, but unlike queens and workers, their activity is not well-known. Drones exit the hive to mate as their only goal, in spite of the high chances of getting lost, being preyed upon and starving; and only if they are lucky, they will die when mating. These nuptial flights take place in Drone Congregation Areas during spring and summer, and we know that their exit time is in the afternoons. Although, drone activity at other hours of the day has not been studied. Using three methods (direct observation, video records and microchip tagging) we evaluated drone activity during the whole day in Northwest Argentina. We detected 24 h of activity for the first time. Surprisingly, several drones were active at dawn, morning, and late morning. Most of them were active in the afternoon, as previously reported. The activity at dawn and during the morning could be a normal pattern in drones around the world, or it could be a result of abnormal factors, such as environmental variables altering their biological clocks.

**Abstract:**

Males in Hymenopteran societies are understudied in many aspects and it is assumed that they only have a reproductive function. We studied the time budget of male honey bees, drones, using multiple methods. Changes in the activities of animals provide important information on biological clocks and their health. Yet, in nature, these changes are subtle and often unobservable without the development and use of modern technology. During the spring and summer mating season, drones emerge from the hive, perform orientation flights, and search for drone congregation areas for mating. This search may lead drones to return to their colony, drift to other colonies (vectoring diseases and parasites), or simply get lost to predation. In a low percentage of cases, the search is successful, and drones mate and die. Our objective was to describe the activity of *Apis mellifera* drones during the mating season in Northwestern Argentina using three methods: direct observation, video recording, and radio frequency identification (RFID). The use of RFID tagging allows the tracking of a bee for 24 h but does not reveal the detailed activity of drones. We quantified the average number of drones’ departure and arrival flights and the time outside the hive. All three methods confirmed that drones were mostly active in the afternoon. We found no differences in results between those obtained by direct observation and by video recording. RFID technology enabled us to discover previously unknown drone behavior such as activity at dawn and during the morning. We also discovered that drones may stay inside the hive for many days, even after initiation of search flights (up to four days). Likewise, we observed drones to leave the hive for several days to return later (up to three days). The three methods were complementary and should be considered for the study of bee drone activity, which may be associated with the diverse factors influencing hive health.

## 1. Introduction

Males of all Hymenoptera—bees, wasps, and ants—are haploid and outbreeding [1]. In social Hymenoptera, they are considered more as a burden to the colony because they must be fed and assisted, hence their name “drone” meaning parasite [2]. In *Apis mellifera*, the mating season occurs during the spring and summer. Males exit from the hive, perform orientation flights, and search for drone congregation areas (DCAs) to try to mate. Although drones have a low probability of success due to competition with hundreds of males from other colonies [3]. This “suicidal” search leads to critical outcomes: getting lost, predation, starvation, desiccation and, if lucky, mating, and death. Drone flights are important because drones may return to other colonies and transmit diseases and parasites, drone flights are important for commercial production of adequately mated queen bees to head colonies. In addition, just the basic understanding of a seasonal and daily activity cycle in nature is important. We ask what makes drones go out to search for DCAs, what external or internal signals exist if any. 

On a global scale, including all *Apis* species and *A. mellifera* races, drone activity begins sometime in the afternoon (Table S1). On seasonal scales, this activity is related to the rise of environmental temperature in spring-summer in both hemispheres, and it is related to the beginning of the mating behavior [4]. Right after emergence, *A. mellifera* drones perform orientation flights, which are short (from 3 to 10 min) and during which drones rise and fall spirally, learning the precise location of their hive in their surrounding [5]. After learning the location of their hive, drones search for DCAs [6], and after doing that they return to their hives or drift to other hives to feed [7,8]. Their orientation and return to the original hive and apiary depend on the characteristics of the landscape, such as cardinal points and slope [9]. The flights in search of DCAs have a longer duration (more than 30 min) than the orientation flights. Once the DCA has been found, *A. mellifera* drones could visit them several times during the mating season. These areas exhibit unique characteristics such as slope orientation and land cover that we continue to characterize further [4,6,9,10,11,12].

Shaped by evolutionary and ecological drivers, the circadian rhythm in animals is critical in regulating processes associated with animals’ seasonal and daily activities, such as foraging and mating [13]. The circadian rhythm in honeybees (*Apis mellifera*) may influence the hive’s survival and persistence [14,15,16]. For instance, workers and drones respond to the circadian clock to time their tasks related to the hive’s maintenance and to reproduction, respectively [14]. It has been shown that nursing bees do not exhibit circadian rhythm in the colony and that the social context, particularly brood pheromones, contribute to the inhibition of this rhythm [14,15,16]. The relationship between circadian rhythm and temperature, for instance, can be confirmed by examining bee brood patterns in relation to internal and external thermal conditions of the hive. The hive’s internal temperature remains constant at ~35 °C in the brood area in the center of the colony [16] and fluctuates at the periphery. The colony temperature is in part influenced by the behavior of drones. Drones perform a thermoregulation function when needed [17]. Immature drones stay within the brood area and mature drones are located in the periphery where the food is stored [18]. In the periphery, the temperature fluctuates more. Since mature drones are the ones that go out to mate, this behavioral preference for the periphery leads to the hypothesis that peripheral oscillations in temperature may be a potential trigger for the endogenous clock of drones.

Different methods have been used to examine bee activities, including direct observations at the hives [19], video recordings [20], radars [21,22], and tags read by different devices, such as QR codes and radio frequency identification (RFID) [23,24,25,26,27]. RFIDs have several advantages over other methods, including the possibility of tracking a bee from birth as well as tracking bees that are under environmental stressors, such as exposure to pesticides or other chemical compounds [20]. The use of RFIDs in the mating season, for instance, has revealed the previously unknown behavior of the queen leaving the hive several times in the season [28]. Most of these methods have been used to study the activities of female bees. By contrast, studies of the detailed activity of drones using RFIDs have not been reported. RFIDs may show new patterns of drone activity that can add to our understanding of the behavioral ecology and the reproductive biology of *A. mellifera*.

The objective of this study was to describe the activity of *A. mellifera ligustica* drones during the mating season in Northwestern Argentina using three different and complementary methods: direct observations, video recordings, and RFID technology. Northwestern Argentina is of special interest because of the hybridization of Africanized and European honeybee races in this region [29]. This region also presents a wide range of environmental conditions, from dry forests in the East to subtropical humid forests in the West. It is an extensive agricultural production area, with soybean, sugar cane, grains, and many citrus crops, in addition to livestock. The land used in agricultural production is expanding, leading to a continuous loss of natural environments with high biodiversity [30]. Our knowledge of bee health and behavior in this scenario is sparse, notably including our poor understanding of the consequences of the loss of natural resources, increased competition within the species and with other species, and an increased exposure to contaminants. In this context, it is critical to understand the mating behavior of drones and identify the areas where they choose to mate (DCAs). These areas represent an opportunity to evaluate honeybee health, especially when the DCAs are located within an agricultural frontier. Drones at DCAs are groups from approximately 200–300 colonies located at a distance of up to five km [5]. The information on drone behavior could strengthen opportunities to establish conservation areas for regional ecotypes of honeybees, since these DCAs are reused and maintained by bees over time. In each reproductive season, the queens and drones meet in these same areas.

Questions we ask in the context of drone activity tracking are: (1) What are the hive arrival and departures times for drones in this region of the world? This is important from both basic and practical perspectives, given that we do not know to what degree drone flight times change across the world and this knowledge would facilitate queen breeding conducted by beekeepers. (2) How many drones exit the hive in the morning and how many in the afternoon? Given that beekeepers reported drone activity in the mornings in this region of the southern hemisphere, we needed to corroborate this information and compare it to what is known for the northern hemisphere. (3) Do drones that leave the hive in the morning also leave the hive in the afternoon? In the northern hemisphere, drone activity occurs only in the afternoon. (4) How many flights per day on average do drones perform during the mating season? Queen breeders could use this information as a base to device practices to increase the flight numbers, such as supplemental feeding of colonies during drone flight season. To answer these questions, we studied experimental hives at an agricultural-subtropical forest frontier in the province of Tucumán, NW Argentina.

## 2. Materials and Methods

As Argentina is located in the southern hemisphere, summer occurs from December to March. The province of Tucumán includes diverse ecoregions. The western part of the province is characterized by the humid subtropical Yungas forest, while the eastern part is characterized by the semiarid Chaco forest. The annual average minimum and maximum temperatures recorded in the province vary between 8 °C in the cold season and 31 °C in the hot season, respectively. During the mating season in the spring (September to November), though, climatic conditions can be extreme in this region of Argentina, with maximum temperatures reaching 35 °C to 42 °C, heavy rains of 14 mm per day, and strong winds of 25 km/h. Climatic data of Tucumán were collected from the Agrometeorology Section of the Obispo Colombres Agro-Industrial Experimental Station (EEAOC) website (http://www.eeaoc.org.ar/agromet/index.php) (accessed on 15 March 2018).

### 2.1. Direct Observations at the Hive’s Entrance

We made direct observations of drone activity at two apiaries in Tucumán. One of the apiaries is located in the vicinity of the subtropical humid forest, at the locality of Horco Molle (26°46′53.93″ S, 65°19′6.94″ W), and the other is located in the drier locality of Leales (27°11′34.56″ S, 65°13′42.15″ W). We collected data in Horco Molle during September and October 2015 and in Leales during September and October 2015, and we repeated the data collection in January and February 2016. We registered the activity of approximately 20 hives every day for two weeks in the spring and again for two weeks in the summer. At the hive entrance, we recorded visually the departure and arrival times of every drone without tags, to determine the hour of highest activity during the day. During the two-week period, visual observations were made between 8 a.m. and 6 p.m., in two-hour periods that changed from one day to the next (e.g., Day 1: from 8 a.m. to 10 a.m.; Day 2: from 10 a.m. to 12 p.m.; and so on). During each two-hour period, observations were made three times in 10-min-intervals (30 min). For the 20 hives in these observations, there were approximately 40 h of observation for both seasons. The observations were performed on days with sunny weather conditions, at the hive entrance with at least two observers collecting data at the same time.

We also obtained video recordings using a digital video camera JVC 40X Full HD, adapted to an additional separate hive with an observation platform at the entrance. This wooden 20 × 40 cm platform was covered with a glass that allowed the recordings of drone activity during the mating season. In this single hive where video recordings were made, we have marked approximately 200 drones with colored numbered tags for individual identification. The drone detection was done visually on a monitor, analyzing recordings frame by frame.

### 2.2. Use of Radio Frequency Identification Tags

**Figure 1:**
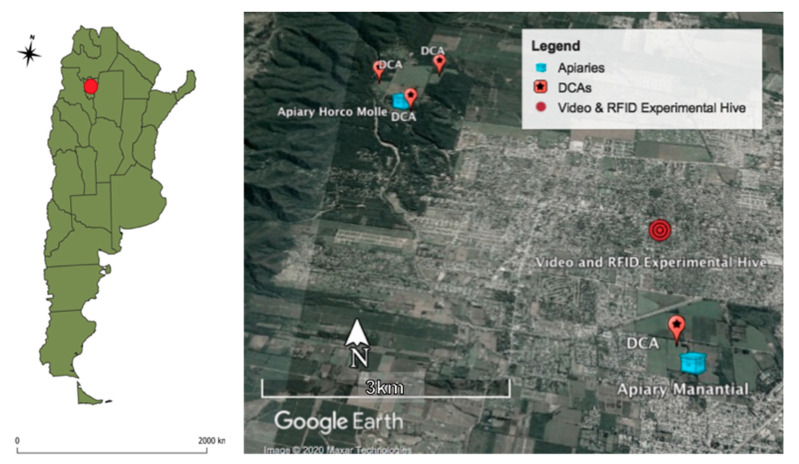
Map of Argentina and Radio-Frequency Identification (RFID) experimental hive in an urban landscape, with two apiaries and four drone congregation areas (DCAs) (Google Earth^®^).

In a second additional hive containing an Italian queen (*A. mellifera ligustica*), we adapted a radio frequency identification and scanning system and attached glued tags (i.e., RFID microchips) to the thorax of the drones [24]. Each microchip included an identifier that allowed discriminating individual drone activity. This second hive was located in an urban landscape, 5.23 km South East of the base apiary in Horco Molle locality (26°49′8.61″ S, 65°17′2.17″ W); 1.91 km North of the nearest DCA (26°50′10.13″ S, 65°16′37.38″ W); and 2.27 km North from the closest apiary of the Agronomy and Zootechnical Faculty of the National University of Tucumán (26°50′21.61″ S, 65°16′58.74″ W) (see map, Figure 1).

**Figure 2:**
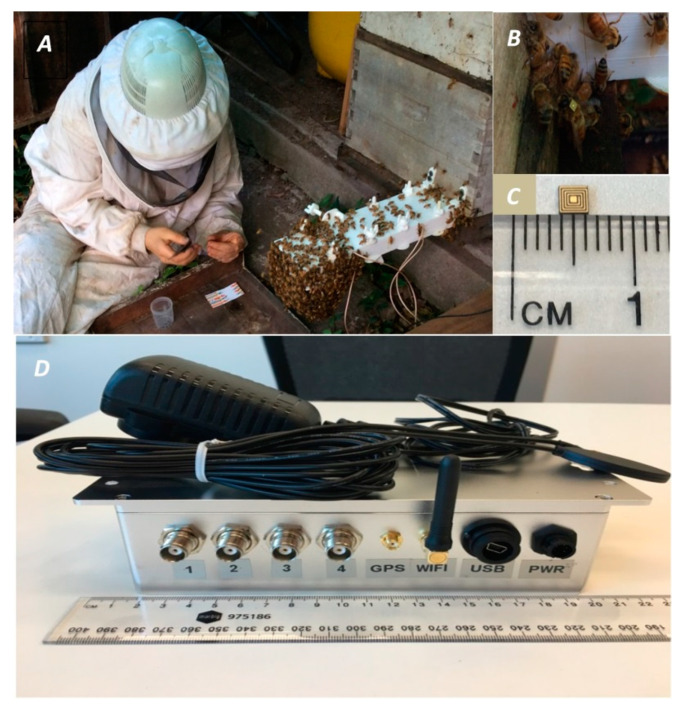
(**A**) Drones being tagged beside the hive. The white box at the entry is where the two pairs of patch-type RFID antennas are located. (**B**) Drone fitted with RFID microchip. (**C**) RFID microchip (Tag). (**D**) Reader unit with four connectors visible (one for each RFID antennas, Wi-Fi antenna, GPS connector and data and power connectors. A full description of this system is provided at [25].

During the mating season, in 15 October 2017, we tagged the drones individually at the hive’s entrance (Figure 2). Before marking them, we collected a sub-sample of drones and extruded their endophallus to verify the association between the softness of drones’ bodies with the color of the cornua [31]. We concluded that soft-bodied drones had uncolored cornua, and thus were immature. Next, based on the hardness of their bodies, we selected only hard-bodied drones to perform the final tagging. The scanner continuously measured the entry and exit of the tagged drones for a month and a half (15 October to 1 December 2017). We tagged 200 drones, approximately 40% of the total drones in the hive. This beehive contained five brood frames and enough food (two frames of pollen), plus a medium honey super (ca. 7 kg of honey).

### 2.3. Departure & Arrival Detection Using RFIDs

“Departure” and “Arrival” records were identified by examining the collected scanner data. A specific sequence of sensor crossings represented either type of movement. A complete “Departure” from the hive was registered when the individual drone tag identifier first crossed sensor A, then crossed sensor B, and afterwards returned to the sensor B (1). An “Arrival” record was registered when the individual drone tag identifier first crossed sensor B, then crossed sensor A, and afterwards returned to the sensor A (2). A complete departure-arrival series required, therefore, the sequence shown in formula (3).

(1) Departure=A → B → B

(2) Arrival=B → A → A

(3) Departure & Arrival Series=A →B→B→A→A

In order to determine the “Departure” and “Arrival” times, we used the timestamp of the middle sensor measurement in each sequence. Morning departures were counted when drones left before 12 p.m. in the daytime. Later departures were counted as afternoon trips.

The time a drone remained inside the hive was the time span between a detected “Arrival” and a renewed “Departure”. The time a drone spent outside the hive was the time span between a recorded “Departure” and a subsequent “Arrival” of the same individual. The time spans obtained were categorized based on Susanto et al. (2018) [32] (Table 1). During the categorization we made the following assumptions:

- If the drones leave and enter the hive frequently, i.e., between 1 s and 3 min, they are exiting to defecate or beard (see Figure 2) and entering to feed.
- If the drones remain inside the hive for more than 30 min, they are resting or waiting for climatic conditions to improve.
- If the drones leave the hive for a period of 3 to 10 min, they are performing orientation flights.
- If the drones leave the hive for a period of 10 min to 10 h, they are searching for or staying at the DCA.
- If the drones are undetected again, they are dead or mated, or disoriented.

### 2.4. Reproducibility & Renewability of the Analysis 

The data analyses were performed in the *R* environment (R Core Team 2017) [33]. Based on the provided *R* codes, the drone activity movement product can be regularly updated by simply replacing the RFID records in the *R* code and re-running the remaining code-parts. 

Please find the R codes in the next link: https://github.com/philippgaertner/article-supporting-info/tree/main/2020-honeybee-activity-analysis. (accessed on 5 January 2008).

## 3. Results

### 3.1. Direct Observations at the Hive’s Entrance and Video Recording

**Figure 3:**
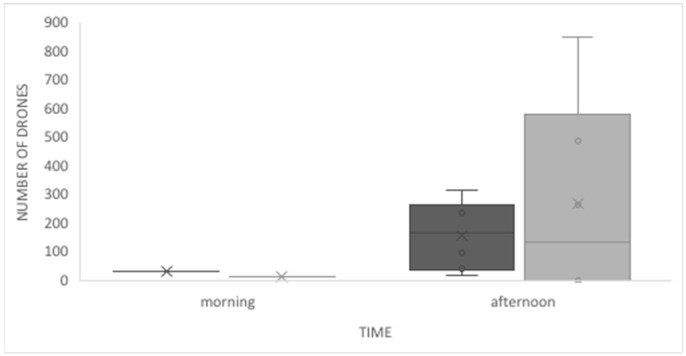
Drones’ flight activity as determined by video recording (dark grey) and direct observations (grey), in the morning and afternoon in Tucumán, NW Argentina.

We found no significant differences between the observations made directly in the hive and those made by video recording (MWW, *U* = 15.5; *p* = 0.059). Similarly, we found no difference between drone activity in the morning and the afternoon, when comparing video recordings and direct observation (MWW, *U* = 23.0; *p* = 0.142; Figure 3). At the observed hives, drones tended to leave the hive at 11 a.m. during the mating seasons of 2015, 2016, and 2017. The highest activity in the morning corresponded to the arrival of drones to the hive. During these morning observations, we often recorded drones arriving but not entering the hive because the workers on duty did not let them in. When they arrived at the entrance, they either returned to flight immediately or took a short rest before flying again, or even stayed at the entrance for up to 30 min. Some drones that were leaving the hive, stayed at the entrance passing their front legs over their eyes and antennas, and then flew. All these behaviors occurred mostly in the mornings, when the flow of drones was small. 

In the afternoons, the behaviors at the entrance changed, given the increased drone activity and the impossibility for the guard bees to stop the flow of drones. The drones leaving the hive did not stop for grooming, they flew immediately; the arriving drones entered the hive erratically. These erratic behaviors included inaccurate landings and/or collision with other bees or with the entrance or the hive box. During the hottest days, the entrance was covered by drones, hindering the workers’ activities. Drones returning to the hive were fed in the entrance by the workers. The activity schedule changed through the seasons of spring and summer in the southern hemisphere; some drones left earlier in the morning in August and September, from 10 a.m. to 11 a.m., at the beginning of spring; and most left in the afternoon, between 2 p.m. to 3 p.m. in October and November. Some of them continued arriving until 6:30 p.m. in all months. In spring (September to November) there was some drone activity also, from 11 a.m. to 12 p.m., but the highest activity was in the afternoon at 3 p.m. In summer, the activity of drones was only in the afternoon, from 3 p.m. to 5:30 p.m.

### 3.2. Scanning with RFIDs

**Figure 4:**
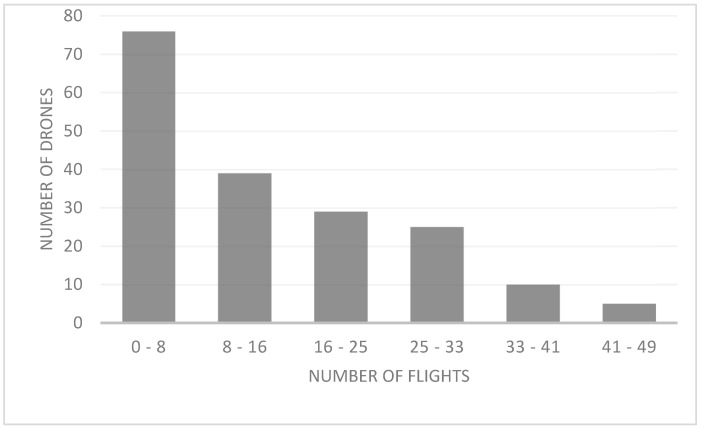
Number of drones and number of flights, by range, during the mating season in NW Argentina.

We found that drones exhibited 24-h activity (i.e., arrivals and departures). From the total of 200 drones tagged, 16 (8%) were not recorded during the experiment, possibly because the chip became unglued, or because drones left without entering the hive, were predated or mated and died. The scanner registered 20 (10%) drones with only one reading. This scanner reading may be because these drones went out looking for a DCA and were successful and died by mating or were unsuccessful and were lost or predated. The average total number of recorded flights per drone was 14.58 (min. = 1; max. = 49), with a maximum of 49 flights registered in three drones. We registered few drones with many flights and many drones with few flights (X^2^ = 346.471; df = 2; *p* < 0.000; Figure 4). 

**Figure 5:**
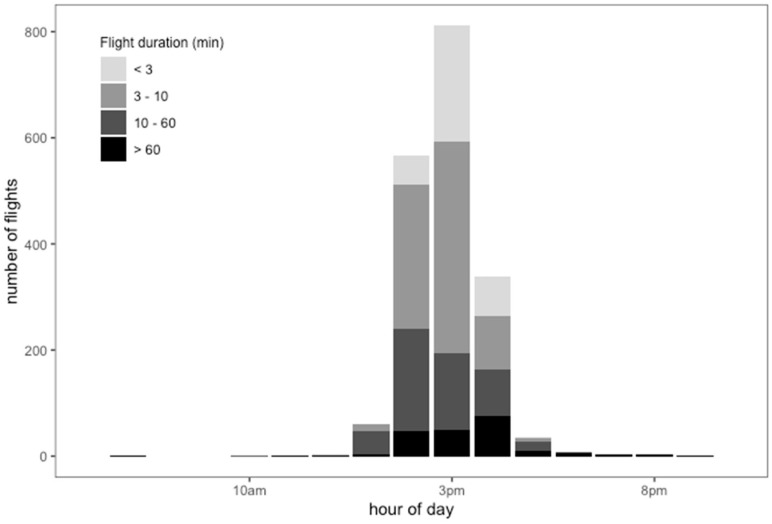
Number of flights according to flight duration in minutes of drones marked with RFID in different hour ranges during the mating season in NW Argentina.

The mean daily number of flights per drone was 12.3 (min. = 6; max. = 28), with a mean duration of 28.05 min (min. = 0.566; max. = 813.35; Figure 5). The RFID data confirmed the activity pattern of drones recorded by direct observations at the hive’s entrance and at the DCAs, specifically showing that activity peaked in the early afternoon (3 p.m.) (Figure 5). We found the drones undertook several flights of different durations, ranging from less than 3 min to more than 60 min according to the hour of the day. The 7.3% of total flights that had the shortest duration (less than 3 min) most often took place between 2 p.m. and 4 p.m. Most common were flights lasting from 3 to 10 min, accounting for 76.22% of total flights. We found that 12.4% of the flights had a duration of 10 to 60 min, and their activity decreased between 1 p.m. and 4 p.m. Flights lasting longer than 60 min, accounting for 4% of the total, were more frequent near 5 p.m. Three- to 10-min activity visibly increased from 1 p.m. to 2 p.m. and decreased from 3 p.m. to 4 p.m. Few drones showed activity lasting longer than 60 min before 12 p.m. or after 6 p.m. Short flight activity and less than 60-min flights were not registered after 5 p.m. Also, activity of longer than 60-min duration fell abruptly at 6 p.m. (Figure 5).

**Figure 6:**
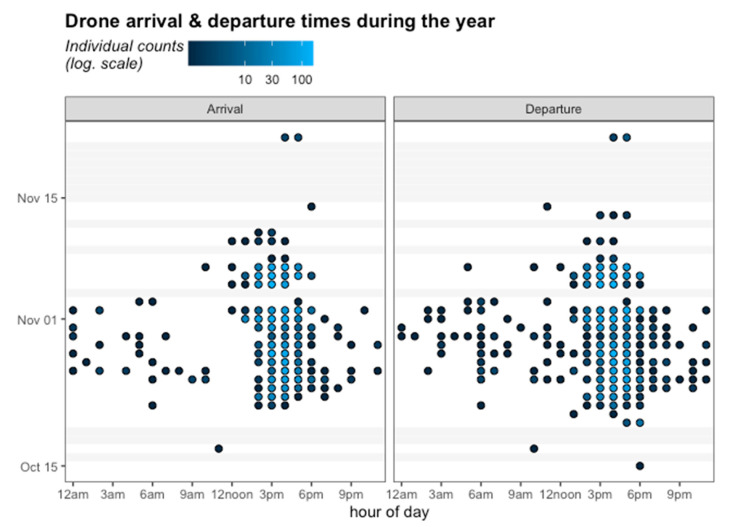
Arrival (**left panel**) and Departure (**right panel**) times of the drones marked with RFID in Tucumán. The color gradient goes from black to blue, where black indicates few individual counts and blue indicates more than 100 counts. Grey bars indicate days without activity.

No departure activity was observed on October 17 or 18, 2017 (see Figure 6), when the temperature was extreme, with a thermal sensation of 42 °C, and clouds were absent. The same occurred on November 3 and 4, 2017, when precipitation was intense (Figure 6). A Pearson correlation analysis between average temperature and average precipitation on the days of the experiment showed a positive (r = 0.153; *p* = 0.35) and a negative (r = −0.156; *p* = 0.34) effect of these climatic variables on the average number of flights daily, respectively, although there was no correlation between temperature and precipitation. 

In addition, the results showed that 21.5% of the tagged drones were recorded leaving the hive in the mornings, and 16% of them showed activity in both the morning and the afternoon. The individual arrival and departure count for drones during the monitoring time showed activity between 3 a.m. and 10 a.m., and in the afternoons more than 100 counts were recorded between 3 p.m. and 6 p.m. After November 10, little activity was recorded and only in afternoons. 

We were able to detect the activity of each drone that departed in the early morning and the late afternoon. We identified drones that departed and arrived up to four times in the early morning on separate days (such as Drone ID: 065 and 125 in Figure S1). One of them departed at dawn and other times of the morning and recorded more than five counts before midday (Drone ID: 065 in Figure S1, see violet points).

**Figure 7:**
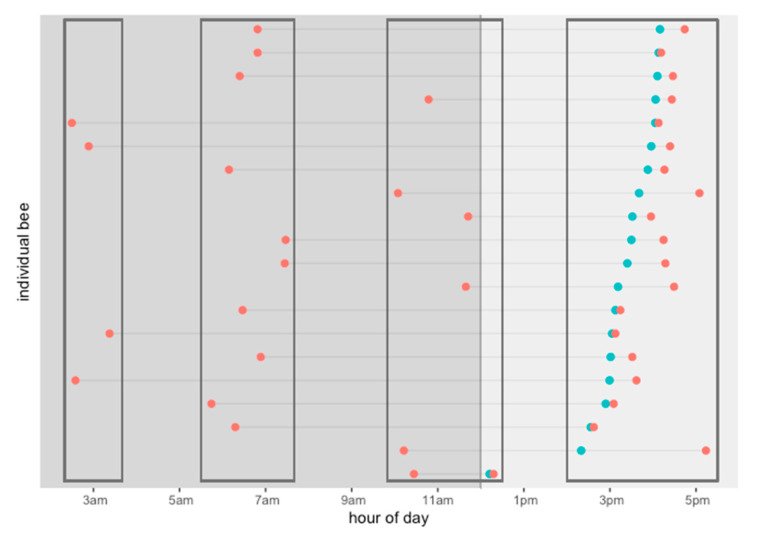
Activity pattern of twenty (20) RFID tagged drones that showed unusual activity times in one day, with flights lasting more than 60 min. They were divided into groups according to their departure time during the day. Departure (red) and arrival (light blue).

Our data show that the daily activity of the drones can be divided into four departure-time groups: at 3 a.m., 6 a.m., 12 p.m., and 5 p.m. (see Figure 7). Some drones departed at dawn, subsequently returned to the hive, and then departed in the mid-morning for a second time. Most of the drones that departed once, returned, and then came out again generally did so between 9 a.m. and 11 a.m. (Figure 7). 

We found 20 drones that showed activity at unusual times for bees in one day (Figure 7). They all made flights lasting longer than 60 min. They left the hive at different times—at dawn and early morning up until before noon—and they all returned all at the time of peak hive activity in the mating season, namely, between 2 p.m. and 6 p.m. (Figure 7). They returned to the hive after a long absence, came in for food, and went out again. We found that between 2 a.m. and 4 a.m., four drones left the hive and returned at 3 p.m. Between 6 a.m. and 8 a.m., ten drones left the hive and returned between 2 p.m. and 6 p.m. Finally, six drones left the hive between 10 a.m. and 12 p.m. and came back between 2 p.m. and 6 p.m. A notable exception was one drone that arrived after 12 p.m. and then exited again quickly (Figure 7).

Several drones remained inside the hive for up to four days, and some others remained for up to two or three days. Some drones remained outside the hive for up to three days. The number of drones outside the hive for 24 h was notably larger than the number of drones outside the hive for two to three days (Figure S2). We had high activity readings of short flights and few readings of drone activity of more than a day outside the hive. 

### 3.3. Comparing Methods

Table 2 summarizes some of the strengths and limitations of the three methods used in this study. 

## 4. Discussion 

This study describes in detail the activity of *A. mellifera ligustica* drones during the mating season, for the first time in Northwestern Argentina, using complementary methods. By means of direct observations at the hive’s entrance, video recordings, and RFID technology, we were able to determine that drones were active throughout a 24-h period. Drones’ arrivals were more easily detected than their departures by direct observations given the longer time the drones take to land versus the shorter time they take to depart. Video records, observations at the DCA (see Data S1), and RFIDs confirmed the pattern of higher drone activity in the afternoons. The data obtained by videography are continuous, and reviewers of the video can use the rewind and fast forward functions to make sure that the image being observed matches the drone that we are looking for.

Some activity was registered by all methods during the mornings in this part of the world. At the DCAs, drones’ visits at this time of the day could be attributed to disorientation in terms of time or the impossibility of entering their original hive (Figure 4 and Figure 6, and Figure S1). Young drones might be coming to the DCA as possible scouts that recognize the area for a later return. Field observations in the DCA (see Data S1), at the beginning of the mating season (August in the southern hemisphere), showed that drones make ascending and descending spiral flights. This is possibly to gather information about the landscape and the location and thus avoid spatial disorientation.

Drones carry magnetite in their bodies, probably for orientation using the earth’s magnetic field during navigation [35]. If drones are using these magnetic signals, it is likely that magnetic changes during the day (i.e., mornings vs. afternoons) contribute to drones’ arrival/departure to and from the DCAs, but these temporary changes may also cause disorientation. In addition, DCAs could have their own characteristic magnetic signal. This hypothesis remains to be tested with the help of a highly sensitive digital field magnetometer. The fact that we have registered drone activity in the early morning (dawning hours) as well as throughout the morning (Figure 7) raises new questions about the type of tasks that drones may be performing during those periods of the day and the relationship between the drones’ circadian rhythm and behavior. These drones that showed activity in the early morning, may be younger ones, that don’t show a defined circadian rhythm. If not, they may be older drones that are suffering an alteration in their biological clock due to virus infections or pesticides contamination. On the other hand, these drones could just be in a different colony or in the field inside of closed flowers. 

The RFID technology allowed us to obtain the drone flight patterns, in terms of flight duration and its frequency in each hour of their activity period (Figure 5). Despite being presented descriptively, this information can be viewed as a baseline. For example, it is likely that flights of more than 60 min are made by the oldest drones. This differentiation of flights and hours could be complemented with the age of the drones in future studies. On the other hand, shorter mating flights (Figure 5) may also be indicative of other health problems, such as parasitism, probably during pupation by the ectoparasitic mite, *Varroa destructor* [5,36]. We found *Varroa* on the drones at the DCA, and their parasitic load depends on the distance to the nearest apiary [12]. *Varroa*, like any parasite or disease, needs high population concentrations to spread, and an apiary is the best place for this [37]. Because wild beehives are scattered in the field, their infestation rate is more variable and differs from what happens in an apiary [12]. Also, the colony collapse disorder may reduce the frequency and length of scouting trips in workers, which may also affect drone scouting activity. These effects could be studied in future researches comparing them with the baseline that we obtained in this work. Likewise, other factors that may affect drone flight, like environmental and hive temperatures, can be studied based on this baseline (Figure 5).

Individual drone’s arrival and departure behavior was also reflected by the RFIDs. We showed for the first time that drones were active throughout the day. In addition, although staying outside the hive increases the probabilities of predation, this study commonly found drones outside the hive for three days. This finding may be a result of drifting, which occurs when a drone spends the night in another hive, instead of its original one. In a previous experiment conducted in Puerto Rico, we found that some drones that had been released four kilometers away from their original hive returned two days later [9]. Drones that were detected only once likely mated and died or they did not come back because they were predated, disoriented, desiccated or starved. This technology was useful to quantify this mating/predation probability, thus validating its potential use in other environments as a comparative tool. 

In general, drones did not remain inside the hive for long, but some spent up to four days without leaving. Inside the hive, adult drones are active before departing, given their need to heat their thorax before flight. This warming is important to maintain the development of the brood inside the hive [38]; therefore, drones that remain longer inside may be fulfilling this thermoregulation need. A long time inside the hive, on the other hand, suggests the possibility that these drones are still immature and remain inside to take advantage of favorable temperatures for development. Drones commonly fly at temperatures above 19 °C [39], and the departure time increases with increasing temperatures up to around 38 °C [39,40]. According to our results (not shown), the temperature range that would favour flight activity (duration and number of flights) is between 24 and 30 °C. Our records on 17 and 18 October 2017, confirmed the upper limit of temperature. Both days were extremely hot (near 42 °C) and no drone activity was registered. Also, we did not observe any drones outside the hives on those hot days, not even with a pheromone lure in the DCA. 

A relationship between the temperature within the hive and the presence of circadian rhythm has been observed in female bees, and likely this may also occur in drones [16]. Previous evidence suggests that if the drones are exposed to low temperatures, they depart earlier in the day than if temperatures are higher [41]. Colony temperature is constant at 35 °C in the brood nest whereas it shows robust daily oscillations at the periphery [42,43]. Foragers are rhythmic and normally found in the peripheral frames while nurses are arrhythmic and typically found at the brood nest, suggesting that daily oscillations of temperature may serve as temporal cue to the foragers [44,45]. It has been observed that immature drones staying inside the hive prefer the hottest zones (sealed breeding) in the hive, and adults prefer the most variable thermic zones, such as the open cells [18]. This difference in the position of the drones may be related to circadian rhythm, namely, those in the periphery may be entrained by the temperature cycles while those in the brood nest are not.

In insects such as Drosophila, changes in temperature change the rhythms of sexual activity, delaying or advancing mating [46,47]. Hamasaka et al. (2010) [48] found that there is a sexual clock in flies. Though flies have two peaks of daily activity, one in the morning and one in the afternoon, those researchers observed that courtship behavior occurs at a particular time of the afternoon. For bees, the mechanisms triggering drones’ and queens’ departure times to mate are not clear. We know that mating behavior only takes place in the afternoon, after the meridian has passed, according to the solar time (See Table S1 and [49,50,51,52,53,54,55,56,57,58,59]). An external environmental signal for drones and the queen, such as the position of the sun at its highest point at noon may be responsible for their departure time. Visual observations remain relevant as they reveal drone traits and behavioral change that simply cannot be captured by RFID technology. For this purpose, the traditional epidemiological observation will remain relevant.

## 5. Conclusions

Our investigation of drone activity in this region of the world showed that while it does take place at dawning and morning, it is less than in the afternoons. This is consistent with the worldwide pattern in which the greatest drone activity is always in the afternoon, with drones starting to leave the hives around 2:00 p.m. and to return after 5:30 p.m. However, we did find drone activity across a 24-h period. Our examination of how many of the study drones exited the hive in the morning and how many in the afternoon revealed that a good number do leave the hive in the morning. Specifically, we detected that 23% of the marked drones went out in the morning and some of them did so repeatedly. For example, one particular drone went out up to five times on different days. Some drones spent up to four days outside the hive, which might be an indicator of disorientation or drifting. Other drones remained inside the hive for up to three days, which could be an indicator of immaturity or resting. Still, other drones came out and did not return, which might suggest reproductive success, predation, or disorientation. We also examined whether drones that leave the hive in the morning also leave in the afternoon. We detected that 10% of the marked drones went out both in the morning and in the afternoon. This morning activity, however, could be explained by drifting, possibly looking for food, or in some cases hives with queen cells that attract nearby drones. We do not think these particular drones go to visit the DCA to mate since we could not detect them in these places in our sampling. Regarding the question of how many flights per day drones perform during the mating season, we found an average of 14.6 flights. Among methodologies, visual observations remain very important for studying honeybee behavior and activity. It is important to continue research that will help us better understand drone behavior during the mating season and apply that knowledge to the tasks of queen breeders.

## Figures and Tables

**Table 1:** Inputs to generate scripts in *R* to illustrate the activity of a drone in a day, including an interpretation of the events associated with the activity of each bee drone.

Drone Behavior	Activities	Threshold	Duration	Frequency
Exit/Entry	High frequency readings, defecation, beards, feeding	x ≤ 3 min	1 sec < x ≤ 3 min	High
Resting	Inside the beehive, e.g., climate	x ≥ 30 min	30 min < x ≤ 10 h	High
Short mission	Orientation flights, walking outside around the hive	3 min < x ≤ 10 min	3 min < x ≤ 10 min	Medium
Scouting	Visiting or scouting for new drone congregation areas	x > 10 min	10 min < x ≤ 10 h	Low-Medium
Departed drone	Last detection of an individual, e.g., drone mating	-	-	High

**Table 2:** Comparison of the strengths and limitations of the three methods used in this research: direct visual observation, indirect visual observation, and electronic tagging (RFID).

Methods	Strengths	Limitations
Direct visual observation at the hive’s entrance	Detailed behavioral observation such as interaction with other drones and workers, e.g., trophallaxis; interaction with other insects and with their parasites, such as phoretic *Varroa.*	Difficult to perform on hot days owing to agglomeration of individuals Time-consuming Depends on human experience Limited nighttime observation
Indirect visual observation (via camera)	Individual marking feasible (limited to markers) Possible to process image to quantify movements Reduced observer error as you can rewind and re-observe	Limited to marking patterns to identify individuals Post-processing could be time-consuming Depends on quality of hardware and software Sufficient illumination is necessary for good results
Electronic tagging (via RFID)	Low cost Easy to install Operates 24 h/day	Extra weight to bees Chemicals (e.g., glues) can have adverse effects on the bees [34]. Some RFID systems can miss readings Reduction of hive entrance size.

## Data Availability

Data available in a publicly accessible repository that does not issue DOIs. https://github.com/philippgaertner/article-supporting-info/tree/main/2020-honeybee-activity-analysis. (accessed on 5 January 2008).

## Supplementary Materials

The following are available online at https://www.mdpi.com/article/10.3390/insects12060566/s1, Table S1: Reported Local Apparent Time (LAT) and Local Mean Time (LMT) activity of the drones of *Apis mellifera* in different locations of the world. Figure S1: Individually tagged drones departing in the mornings, during the nuptial flight season. Figure S2: Activity of all RFID tagged drones expressed as the number of days that each drone remained inside and outside the hive during the mating season. Data S1: Methodology and Observations at the drone congregation areas.
