# Supplementary material for: Analysis of Honeybee Drone Activity during the Mating Season in Northwestern Argentina
Source: Insects. 2021 Jun 21;12(6):566. doi: 10.3390/insects12060566 (PMC8234112; doi:10.3390/insects12060566)
Supplement: Supplementary file 1 [file insects-12-00566-s001.zip › insects-1206368-SI.pdf]

Analysis of Honeybee Drone Activity During the Mating Season in Northwestern Argentina

Supplementary Material

Table S1. Reported Local Apparent Time (LAT) and Local Mean Time (LMT) activity of the drones of *Apis mellifera* in different locations of the world.

		Legal time	Solar time		
**Location**	**Place**	**Local Apparent Time (LAT)**	**Local Mean Time (LMT)**	**Difference in minutes**	**Reference**
7.87N- 80.77E	Sri Lanka	12:45 pm - 03:45 pm	12:54 pm - 03:54 pm	9'	[49]
36.78N- 119.41W	USA- California	02:00 pm - 04:30 pm	12:40 pm -03:10 pm	80'	[50]
30.98N- 91.96W	USA- Louisiana	02:00 pm - 03:00 pm	1:10 pm - 02:10 pm	50'	[51] [52]
39N- 98.48W	USA- Kansas	01:30 pm - 05:10 pm	12:15 pm - 04:55 pm	75'	[42]
52.13N- 5.3E	Netherlands	12:00 pm - 04:30 pm	10:40 am - 03:10 pm	80'	[53]
46.2N- 2.21E	France	02:00 pm - 06:00 pm	12:23 pm – 04:23 pm	97'	[21]
51.16N- 10.4E	Germany	12:00 pm - 05:00 pm	11:00 am - 04:00 pm	60'	[28]
47.5N- 14.55E	Austria	02:00 pm - 05:00 pm	01:50 pm - 04:50 pm	10'	[54]
31.04N- 34.85E	Israel	01:00 pm - 04:00 pm	12:35 pm - 03:35 pm	25'	[55]
18.2N- 66.6W	Puerto Rico	02:30 pm - 05:30 pm	02:30 pm - 05:30 pm	0'	[10]
30.41S- 63.6W	Argentina	01:00 pm - 05:30 pm	12:00 pm - 04:30 pm	60'	[56]
6.42N- 66.6W	Venezuela	12:30 pm - 07:30 pm	12:20 pm - 06:20 pm	10'	[57]
30.56S- 22.9E	S. Africa	12:30 pm - 04:00 pm	12:25 pm - 03:55 pm	5'	[58]
30.56S- 22.9E	S. Africa	01:34 pm - 04:34 pm	01:29 pm - 04:29 pm	5'	[59]

References to Table S1

10. Galindo-Cardona, A.; Monmany, C.; Moreno-Jackson, R.; Rivera-Rivera, C.; Huertas-Dones, C.; Caicedo-Quiroga, L.; Giray, T. Landscape analysis of drone congregation areas of the honey bee, *Apis mellifera. J. Insect Sci.* **2012**, *2*, 122.

21. Loper, G.; Wolf, W.; Taylor, O. Honey bee drone flyways and congregation areas: Radar observations. *J. Kans. Entomol. Soc.* **1992**, *65*, 223–230.

28. Heidinger, I.M.M.; Meixner, M.D.; Berg, S.; Büchler, R. Observation of the mating behavior of honey bee (*Apis mellifera L.*) queens using radio-frequency identification (RFID): Factors influencing the duration and frequency of nuptial flights. *Insects* **2014**, *5*, 513–527, doi:10.3390/insects5030513.

42. Giannoni-Guzmán, M.A.; Rivera, E.; Aleman-Rios, J.; Melendez, A.M.; Moreno, M. Perez Ramos, D.L.; Moore, D.; Giray, T.; Agosto-Rivera, J.L. The role of colony temperature in the entrainment of circadian rhythms of honey bee foragers. *bioRxiv* **2020**, doi:10.1101/2020.08.17.254722.

49. Koeniger, N.; Wijayagunasekera, H.N.P. Time of drone flight in the three asiatic honeybee species (Apis cerana, Apis florea, Apis dorsata). *J. Apic. Res.* **1976**, *15*, 67–71.

50. Howell, D.E.; Usinger, R.L. Observations on the flight and length of life of drone bees. *Ann. Entomol. Soc. Am.* **1933**, *26*, 239–246.

51. Oertel, E. Mating flights of queen bees. *Glean. Bee Cult.* **1940**, *68*, 292–293.

52. Oertel, E. Observations on the flight of drone honey bees. *Ann. Entomol. Soc. Am.* **1956**, *49*, 497–500.

53. Minderhoud, A. Recent research. Bee World. **1932**, *13*, 66.

54. Koeniger, N.; Hemmling, C.; Yoshida, T. Drones as sons of drones in Apis mellifera. *J. Apic. Res.* **1989**, *20*, 391–394.

55. Lensky, Y.; Cassier, P.; Nutkin, M. Pheromonal activity and fine structure of the mandibular glands of the honeybee drones (Apis mellifera L.) (Insecta, Hymenoptera, Apidae). *J. Insect Phys.* **1985**, *31*, 265–276, doi:10.1016/0022-1910(85)90002-2.

56. Galindo-Cardona, A.; Quiroga, O.B.; Bianchi, E.; Ayup, M.M. Primer reporte de un área de congregación de zánganos de Apis mellifera (Hymenoptera: Apidae) de Argentina. *Rev. Soc. Entomol. Arg.* **2017**, *76*, 50–53, doi:0.25085/rsea.761207.

57. Hellmich, R.L.; Rinderer, T.E.; Danka, R.G.; Collins, A.M.; Boykin, D.L. Flight times of Africanized and European honey bee drones (Hymenoptera: Apidea). *J. Econ. Entomol.* **1991**, *84*, 61–64.

58. Muerrle, T.M.; Hepburn, H.R.; Radloff, S.E. Experimental determination of drone congregation areas for *Apis mellifera capensis* Esch. *J. Apic. Res.* **2007**, *46*, 154–159.

59. Jordan, L.A.; Allsopp, M.H.; Oldroyd, B.P.; Wossler, T.C.; Beekman, M. A Scientific note on the drone flight time of *Apis mellifera capensis* and A. scutellata. *Apidologie* **2007**, *38*, 436–437.

Figure S1. Individually tagged drones departing in the mornings, during the nuptial flight season. Each color indicates how many times each drone departed from the hive. Red = one time, green = two times, blue = three times, and violet = five times.

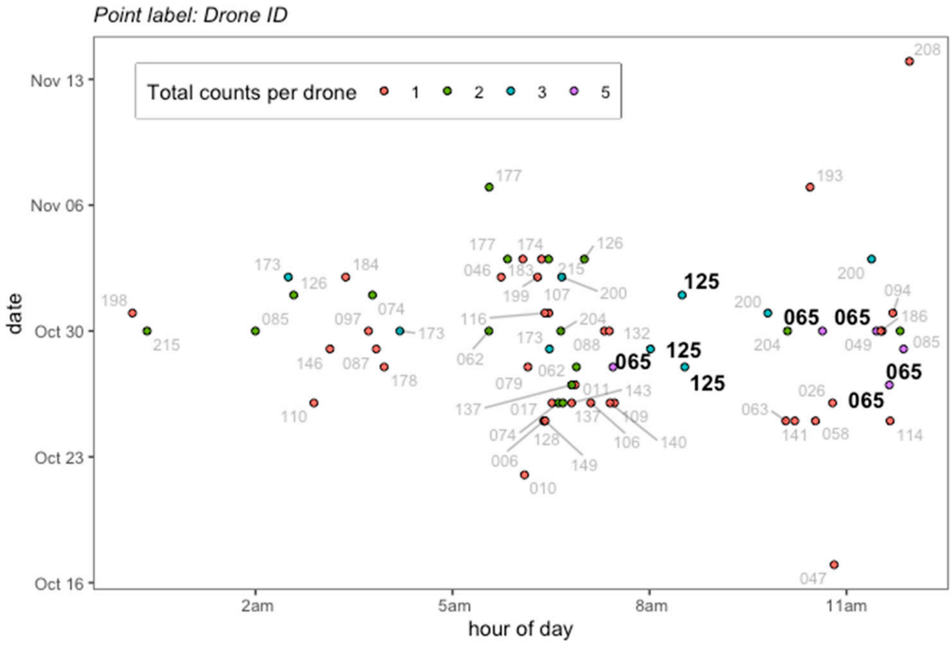

Figure S2. Activity of all RFID tagged drones expressed as the number of days that each drone remained inside and outside the hive during the mating season.

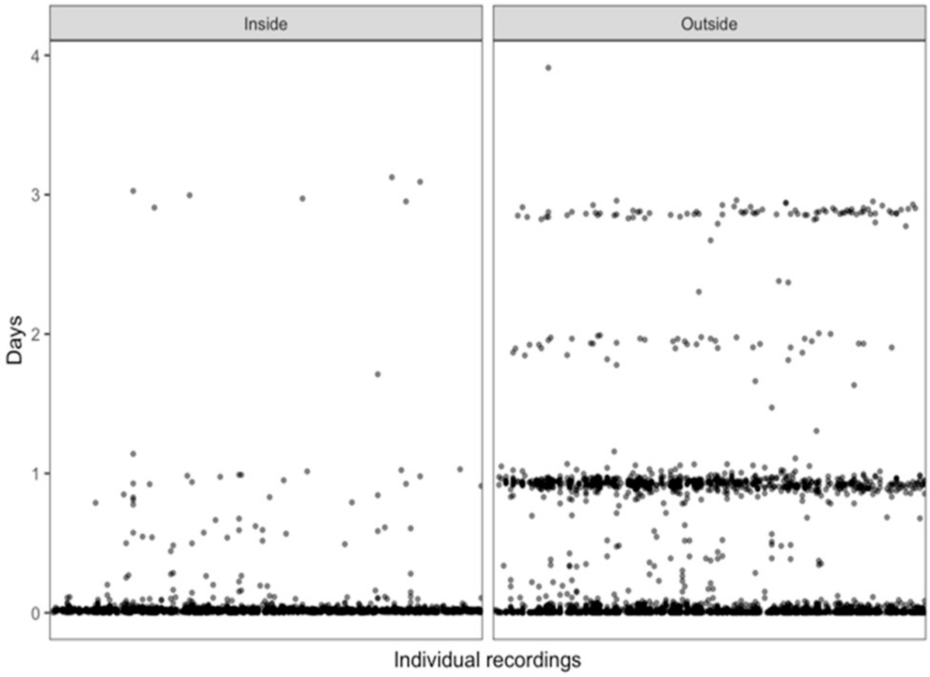

Data S1

Methodology and Observations at the drone congregation areas

Using helium balloons with a synthetic queen pheromone (9-ODA) [see 59, 60], we identified one DCA (26°50'10.13"S, 65°16'37.38"W) near the apiary of the Agronomy and Zootechnical Faculty of the National University of Tucumán and three DCAs near the apiary of Horco Molle (a: 26°47'32.97"S, 65°19'25.72"W; b: 26°47'6.13"S, 65°19'49.98"W; c: 26°50'7.78"S, 65°17'4.06"W) (Figure 1).

We made direct observations in the mornings, between 10 a.m. and 11 a.m., at the DCAs and found low drone activity (e.g., one or two drones attracted by the synthetic pheromone) during this

time. In the afternoons, however, the activity increased significantly from hundreds to thousands. This is an estimated number based on the direct observations of drones following the synthetic pheromone attached to a balloon at DCAs for other projects [12].

Reference to Data S1

12. Galindo-Cardona, A.; Scannapieco, A.C.; Russo, R.; Escalante, K.; Geria, M.; Lepori, N.; Ayup, M.M.; Muntaabski, I.; Liendo, M.C.; Landi, L.; et al. Varroa destructor parasitism and genetic variability at honey bee (Apis mellifera) drone congregation areas and their associations with environmental variables in Argentina. Front. *Ecol. Evol.* **2020**, *8*, 394.
